# Longitudinal Changes in Left Ventricular Structure and Function During a Dietary Weight-Management Trial in Adults with Heart Failure, Type 2 Diabetes, and Obesity: A Secondary Analysis of Pro-HEART

**DOI:** 10.3390/nu18193238

**Published:** 2026-10-01

**Authors:** Lorraine S. Evangelista, Rebecca Meraz, Alona D. Angosta, Angelina P. Nguyen, Kelly L. Wierenga, Jennifer Kawi, Michele A. Hamilton, Gregg C. Fonarow

**Affiliations:** 1 Sue & Bill Gross School of Nursing, University of California Irvine, Irvine, CA 92697, USA; 2 Louise Herrington School of Nursing, Baylor University, Dallas, TX 75246, USA; 3 Cizik School of Nursing, The University of Texas Health Science Center at Houston, Houston, TX 77030, USA; 4 Smidt Heart Institute, Cedars-Sinai Medical Center, Los Angeles, CA 90048, USA; 5 Ahmanson-UCLA Cardiomyopathy Center, Division of Cardiology, David Geffen School of Medicine, University of California, Los Angeles, Los Angeles, CA 90095, USA

**Keywords:** heart failure, obesity, left ventricular remodeling, nutrition intervention, echocardiography, reverse remodeling, secondary analysis

## Abstract

**Background:** Obesity is associated with adverse cardiac remodeling in heart failure (HF), but changes during dietary weight management are not well described. We examined longitudinal changes in left ventricular (LV) structure and systolic function during a dietary weight-management intervention. **Methods:** Pro-HEART participants were randomized to high-protein or standard-protein hypocaloric diets. LV end-diastolic dimension (LVEDD), ejection fraction (LVEF), and posterior wall thickness (LVPWT) were assessed at baseline, 3 months, and 15 months. Linear mixed-effects models tested time and diet effects. **Results**: At 15 months, LVEDD data were available for 31 of 92 participants (33.7%). Achieved protein intake was similar between randomized groups. Among 92 participants, LVEDD changed significantly over time (F(2, 51.20) = 7.45, *p* = 0.001), with model-estimated mean LVEDD decreasing from 59.00 mm at baseline to 54.56 mm at 15 months. LVEDD decreased from 3 to 15 months (−4.42 mm; 95% CI, −7.52 to −1.33; nominal *p* = 0.006; Holm-adjusted *p* = 0.012) and from baseline to 15 months (−4.44 mm; 95% CI, −6.86 to −2.02; nominal *p* < 0.001; Holm-adjusted *p* = 0.002). LVEF did not change significantly over time (overall *p* = 0.074); an exploratory baseline-to-15-month contrast was +4.11 percentage points (95% CI, 0.55–7.67; Holm-adjusted *p* = 0.072). LVPWT did not change significantly, and no diet group × time interactions were observed. **Conclusions:** LVEDD was lower at longer-term follow-up. LVEF did not change significantly over time, although an exploratory baseline-to-15-month contrast suggested an increase. Because both groups received hypocaloric diets, there was no non-dietary control group, and longitudinal HF medication data were unavailable, the observed cardiac changes cannot be attributed specifically to weight loss or the dietary intervention.

## 1. Introduction

Obesity is common in patients with heart failure (HF) and is associated with changes in cardiac structure and function [1]. Excess adiposity can increase hemodynamic stress and is also linked with hypertension, metabolic dysfunction, inflammation, and altered adipokine signaling, all of which may contribute to left ventricular (LV) remodeling [1]. Observational studies have reported greater LV mass and abnormal ventricular geometry in people with obesity [2], and visceral and epicardial fat may further contribute to adverse remodeling through inflammatory and metabolic pathways [1]. These changes matter clinically because adverse LV remodeling is associated with HF progression and poorer outcomes. In contrast, favorable remodeling has been linked with better treatment-related outcomes in patients with LV systolic dysfunction [3].

Intentional weight loss has therefore emerged as a potential strategy for modifying obesity-related cardiac abnormalities. Studies of substantial weight loss following bariatric surgery have demonstrated reductions in LV mass and end-diastolic volume and, in some patients, a shift toward more normal ventricular geometry [4]. More recently, pharmacologic treatments for obesity have provided additional evidence that favorable changes in cardiac structure may accompany weight reduction. In the STEP-HFpEF echocardiography substudy, semaglutide attenuated left atrial remodeling and right ventricular enlargement, although significant changes in LV dimensions, mass, or systolic function were not observed [5]. These findings support the growing interest in cardiac remodeling as a physiologic outcome of intentional weight reduction in HF, while also suggesting that remodeling responses may differ by HF phenotype, intervention, and cardiac measure examined.

Despite this growing evidence, much less is known about cardiac remodeling during dietary weight-loss interventions in adults with established HF. Dietary management has long been part of HF care. Still, evidence for dietary interventions in patients with established HF remains limited, and the effects of structured dietary weight loss on cardiac structure and remodeling have been examined far less extensively than pharmacologic or surgical approaches [6,7]. Moreover, contemporary studies examining intentional weight loss and cardiac structure have largely focused on bariatric surgery or anti-obesity pharmacotherapy, particularly in HF with preserved ejection fraction. Consequently, there remains a need to better understand whether favorable changes in cardiac structure and systolic function may also occur during structured dietary weight management, particularly among adults with chronic HF across a broader range of systolic function.

The Pro-HEART trial provides an opportunity to examine this question using serial echocardiographic data collected during a randomized dietary weight-loss intervention and longer-term follow-up. Participants with chronic HF, type 2 diabetes mellitus (T2DM), and overweight/obesity were randomized to high-protein or standard-protein weight-loss diets, with echocardiographic assessments obtained at baseline, 3 months, and 15 months.

This study was conducted to characterize longitudinal changes in LV structure and systolic function during a dietary weight-management intervention. Specifically, we examined LV end-diastolic dimension (LVEDD), left ventricular ejection fraction (LVEF), and LV posterior wall thickness (LVPWT), and explored whether changes in LV chamber size were associated with changes in systolic function.

## 2. Materials and Methods

### 2.1. Study Design and Participants

The present study used longitudinal data from the Pro-HEART randomized controlled trial (ClinicalTrials.gov identifier: NCT01423266), which compared high-protein and standard-protein weight-loss diets in adults with chronic HF, T2DM, and overweight or obesity. The trial design, intervention procedures, and primary outcomes have been reported previously [8,9]. Pro-HEART randomized 92 participants between 2009 and 2014 to the high-protein (n = 41) or standard-protein (n = 51) hypocaloric dietary intervention. The parent protocol specified adiposity, cardiac structure (assessed by LVEDD), and functional status as the primary outcome domains [8]. In the previously published 3-month analysis of 76 participants who completed the intensive intervention, mean weight decreased by 3.6 kg in the high-protein group and 2.9 kg in the standard-protein group, with no significant between-group difference in weight loss [9]. All 92 randomized participants had baseline echocardiographic data and were included in the present cardiac-remodeling analysis; no participants were excluded for missing baseline echocardiograms.

Recruitment for Pro-HEART began in August 2009 [8]. Baseline visits in the archived analytic dataset extended through July 2014, and participants were followed for up to 15 months. Participants with chronic HF were recruited from outpatient cardiology clinics in Southern California. Eligibility required optimized HF medical therapy that had been stable for at least 3 months before enrollment; additional eligibility criteria are described previously [8]. Baseline and longitudinal HF medication data, HF etiology, and duration of HF were not available in the archived analytic dataset used for this secondary analysis.

The present analytic cohort included 92 participants assigned to the high-protein (n = 41) or standard-protein (n = 51) dietary intervention. All 92 had baseline echocardiographic data and were included in the present analysis; all available measurements at 3 and 15 months were included in the longitudinal models. The previously published 3-month Pro-HEART dietary analysis included 76 participants [9] and therefore represents a different analytic sample from the present longitudinal echocardiographic analysis.

Race and ethnicity were self-reported. For descriptive analyses, participants were categorized as Black, White, Asian/Other, or Hispanic, consistent with prior Pro-HEART analyses. Asian/Other included participants originally coded as Asian, Native Hawaiian, Mixed, or Other because of small cell sizes [10].

All participants provided written informed consent. The study was approved by the Institutional Review Boards of the University of California, Irvine (HS 2010-7972; 12 March 2012), and the University of California, Los Angeles (IRB#10-000471-AM-00009; 28 November 2011), and was conducted in accordance with the Declaration of Helsinki.

### 2.2. Randomization and Dietary Intervention

After baseline assessment, participants were randomized to a high-protein or standard-protein diet using an allocation algorithm developed by the study biostatistician and balanced by study site, age, sex, race/ethnicity, and baseline exercise capacity.

Both diets were hypocaloric. The high-protein diet provided approximately 30% of energy from protein, 40% from carbohydrate, and 30% from fat; the standard-protein diet provided 15%, 55%, and 30%, respectively [8,9]. Participants received individualized 1200- or 1500-kcal/day meal plans designed to produce a 500–800 kcal/day energy deficit and weight loss of approximately 0.5–1.0 kg/week [8].

Formal 3-day food records were collected during the intensive 3-month intervention phase at the 2-, 4-, 8-, and 12-week assessments. During the subsequent maintenance phase, dietary patterns and weight-management challenges were reviewed at follow-up visits, but formal 3-day food records were not collected. A registered dietitian provided counseling at baseline and 2, 4, 8, and 12 weeks during the intensive 3-month weight-loss phase. Dietary records and food checklists were reviewed at these visits, and both groups were encouraged to engage in regular physical activity [9]. Dietary intake was assessed using 3-day food records collected before follow-up visits and analyzed using the Nutrition Data System for Research [11]. For the present analysis, achieved protein intake during the intensive phase was summarized across each participant’s available dietary records by randomized group.

After 3 months, participants were instructed to continue their assigned diet during a 12-month maintenance period, with follow-up visits at 6, 9, 12, and 15 months. Major study assessments occurred at baseline, 3 months, and 15 months. Body weight was measured at each study assessment using standardized procedures described previously [9].

### 2.3. Echocardiographic Assessment

Two-dimensional and Doppler echocardiography was performed at baseline, 3 months, and 15 months according to the standardized Pro-HEART protocol [8]. Participants were examined at rest in the supine position, and images were obtained using standard parasternal long- and short-axis and apical four-chamber, two-chamber, and long-axis views [8]. The archived study documents do not specify whether M-mode or two-dimensional linear measurements were used to obtain the recorded LVEDD values, nor do they identify the method used to calculate LVEF (e.g., biplane Simpson, Teichholz, or visual estimation). Echocardiograms were interpreted by one designated cardiologist at each study site rather than by a central core laboratory. Readers were blinded to randomized dietary assignment; whether they were blinded to assessment time point was not documented. Formal intra- and inter-observer reproducibility estimates were not available in the archived study records. Echocardiographic values entered into the study database were based on these cardiologist readings.

Echocardiographic measures included cardiac chamber dimensions, systolic and diastolic function, valvular function, and estimated right-sided pressures. The present analysis focused on LV end-diastolic dimension (LVEDD), left ventricular ejection fraction (LVEF), and LV posterior wall thickness (LVPWT), using terminology consistent with contemporary ASE/EACVI chamber-quantification recommendations [12]. The original Pro-HEART protocol prospectively designated LVEDD as the primary measure of cardiac structure [8]; in this secondary analysis, LVEF and LVPWT served as complementary measures of systolic function and LV structure, respectively.

### 2.4. Statistical Analysis

Participant characteristics and echocardiographic measures were summarized using means and standard deviations or frequencies and percentages, as appropriate. Longitudinal changes in LVEDD, LVEF, and LVPWT were evaluated using linear mixed-effects models with categorical time, randomized dietary group, and the group × time interaction as fixed effects. Models were estimated using restricted maximum likelihood, with denominator degrees of freedom calculated using the Satterthwaite approximation. Alternative covariance structures were compared using Akaike’s information criterion (AIC). For LVEDD, an unstructured within-participant covariance structure provided substantially better fit than the random-intercept model (AIC 1262.70 vs. 1288.26) and was therefore retained. For LVEF (AIC 1363.86 vs. 1364.48) and LVPWT (AIC 709.80 vs. 711.47), the unstructured models provided little improvement in fit, and the more parsimonious random-intercept models were retained. LVEDD was the primary cardiac remodeling outcome; LVEF and LVPWT were secondary outcomes.

The parent Pro-HEART trial protocol [8] prospectively specified comparisons of outcomes from baseline to 3 months, baseline to 15 months, and 3 to 15 months. The present focused echocardiographic analysis was not separately prespecified in the trial protocol or registration and was conducted as a post hoc secondary analysis of the existing Pro-HEART data. Accordingly, these protocol-defined time-point contrasts were applied to the cardiac outcomes examined in the present analysis. Estimated mean differences and 95% confidence intervals were reported. For the three phase-specific contrasts within each echocardiographic outcome, *p*-values were adjusted for multiple comparisons using the Holm procedure; nominal and Holm-adjusted *p*-values are reported. Exploratory Pearson correlations examined associations between changes in body weight and LVEDD and between changes in LVEDD and LVEF from baseline to 3 months and from baseline to 15 months. Spearman correlations were used as sensitivity analyses for the change-score associations. As a sensitivity analysis, LVEDD was indexed to body surface area, and baseline-to-15-month change in indexed LVEDD was examined among participants with paired measurements. Body surface area was calculated using the Mosteller formula.

Mixed-effects models included all available repeated measurements and assumed that missing observations were missing at random conditional on observed data. To examine potential attrition bias, baseline characteristics were compared between participants with and without 15-month LVEDD measurements using standardized differences. As an additional sensitivity analysis addressing potential attrition bias, the LVEDD and LVEF models were refit adjusting for sex, race/ethnicity, baseline BMI, and baseline LVPWT, which showed baseline differences according to 15-month LVEDD availability. The LVEDD model was also repeated among participants with 15-month measurements to assess the consistency of the primary findings. To assess sensitivity to departures from the missing-at-random assumption, a delta-adjusted pattern-mixture analysis was conducted for the change in LVEDD from baseline to 15 months. Missing 15-month LVEDD values were multiply imputed using baseline variables associated with follow-up availability, including sex, race/ethnicity, randomized dietary group, body mass index, baseline LVEDD, and baseline LVPWT. Imputed values were then shifted upward by 1, 2, and 3 mm to represent progressively less favorable outcomes among participants with missing follow-up measurements. All tests were two-sided, with *p* < 0.05 considered statistically significant. Analyses were conducted using IBM SPSS Statistics, version 27.0 (IBM Corp., Armonk, NY, USA) [13].

## 3. Results

### 3.1. Participant Characteristics

The analysis included 92 participants with chronic HF, T2DM, and overweight or obesity. Mean age was 58.5 ± 9.7 years, 30.4% were female, and mean baseline LVEF was 38.7 ± 12.7%. Baseline characteristics, including comparisons according to availability of 15-month LVEDD measurements, are presented in Table 1.

Among 39 participants with usable intensive-phase 3-day food records (high-protein, n = 18; standard-protein, n = 21), participants contributed a mean of 2.17 ± 0.86 and 2.19 ± 0.87 records, respectively. Mean energy intake was 1680.7 ± 653.7 kcal/day in the high-protein group and 1394.7 ± 504.8 kcal/day in the standard-protein group, compared with prescribed 1200- or 1500-kcal/day meal plans. Mean protein intake was 80.8 ± 20.6 and 74.7 ± 24.8 g/day, respectively. Protein intake relative to body weight was available for 38 participants and averaged 0.77 ± 0.27 g/kg/day in the high-protein group and 0.70 ± 0.16 g/kg/day in the standard-protein group. Percentage of energy from protein was available for 31 participants (high-protein, n = 14; standard-protein, n = 17) and averaged 23.2 ± 8.7% and 23.8 ± 4.0%, respectively.

Body weight changed significantly over time (F(2, 128.35) = 20.19, *p* < 0.001). Model-estimated mean weight decreased from 108.90 kg at baseline to 105.87 kg at 3 months (estimated change, −3.03 kg; 95% CI, −4.01 to −2.05; *p* < 0.001) and subsequently increased to 108.48 kg at 15 months. The baseline-to-15-month difference was not significant (−0.41 kg; 95% CI, −1.78 to 0.96; *p* = 0.551), and the diet group × time interaction was not significant (*p* = 0.062). BMI showed a similar pattern, with a significant overall time effect (F(2, 128.40) = 21.22, *p* < 0.001). Model-estimated mean BMI decreased from 37.08 kg/m^2^ at baseline to 36.08 kg/m^2^ at 3 months and increased to 36.93 kg/m^2^ at 15 months. The baseline-to-15-month difference was not significant (−0.16 kg/m^2^; 95% CI, −0.60 to 0.29; *p* = 0.489), and the diet group × time interaction was not significant (*p* = 0.059).

Model-estimated weights in the high-protein group were 106.13, 102.55, and 106.80 kg at baseline, 3 months, and 15 months, respectively, compared with 111.66, 109.19, and 110.17 kg in the standard-protein group. Corresponding BMI estimates were 36.42, 35.21, and 36.59 kg/m^2^ in the high-protein group and 37.74, 36.94, and 37.26 kg/m^2^ in the standard-protein group. Weight data were available for all 92 participants at baseline and for 3 months and for 40 participants at 15 months. At 3 months, 17 of 92 participants (18.5%) had lost ≥5% of baseline body weight; at 15 months, 4 of 40 participants with available weight data (10.0%) had lost ≥5% of baseline body weight.

**Figure 1:**
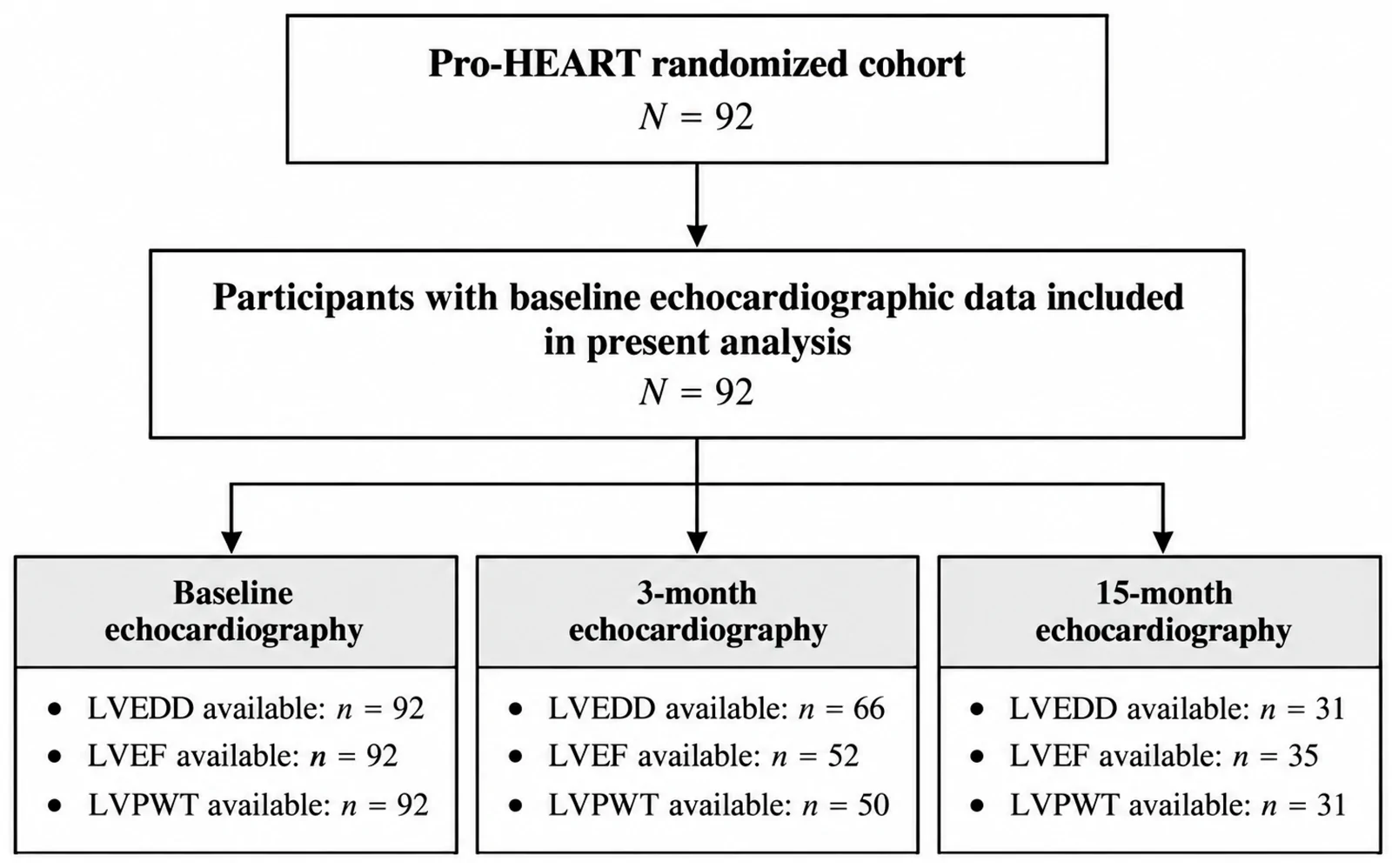
Availability of Echocardiographic Measures Across Study Assessments. Availability was determined separately for each echocardiographic measure. Reasons for missing individual measures were not documented in the archived analytic dataset. LVEDD = left ventricular end-diastolic dimension; LVEF = left ventricular ejection fraction; LVPWT = left ventricular posterior wall thickness.

Participant flow and measure-specific echocardiographic availability are shown in Figure 1. LVEDD was available for 92, 66, and 31 participants at baseline, 3 months, and 15 months, respectively; corresponding numbers were 92, 52, and 35 for LVEF and 92, 50, and 31 for LVPWT. Reasons for measure-specific missingness were not documented in the archived analytic dataset. Standardized differences suggested baseline differences between participants with and without 15-month LVEDD measurements, particularly for sex, race/ethnicity, randomized dietary assignment, body mass index, and selected echocardiographic measures.

### 3.2. Longitudinal Changes in Left Ventricular Structure and Function

LVEDD, the primary cardiac remodeling outcome, changed significantly over time (F(2, 51.20) = 7.45, *p* = 0.001). Neither the dietary group effect (F(1, 87.56) = 1.14, *p* = 0.289) nor the group × time interaction (F(2, 51.20) = 0.47, *p* = 0.627) was significant. LVEDD did not change from baseline to 3 months (EMD, −0.02 mm; 95% CI, −1.51 to 1.47; nominal *p* = 0.982; Holm-adjusted *p* = 0.982). LVEDD decreased from 3 to 15 months (EMD, −4.42 mm; 95% CI, −7.52 to −1.33; nominal *p* = 0.006; Holm-adjusted *p* = 0.012) and from baseline to 15 months (EMD, −4.44 mm; 95% CI, −6.86 to −2.02; nominal *p* < 0.001; Holm-adjusted *p* = 0.002). The diet group × time interaction was not significant (*p* = 0.627). The absence of archived information on the exact LV measurement technique and reader reproducibility is particularly relevant because the observed baseline-to-15-month LVEDD difference was modest and may, in part, reflect measurement variability. In the sensitivity analysis restricted to the 31 participants with 15-month LVEDD measurements, the baseline-to-15-month reduction was similar in direction and magnitude to the primary analysis (estimated difference, −4.32 mm; 95% CI, −7.52 to −1.13; *p* = 0.009).

In the delta-adjusted sensitivity analysis, the baseline-to-15-month LVEDD reduction remained significant when imputed values for participants with missing 15-month measurements were shifted upward by 1 mm (−3.40 mm; 95% CI, −6.22 to −0.58; *p* = 0.018). With a 2-mm shift, the estimated reduction was attenuated and no longer statistically significant (−2.73 mm; 95% CI, −5.55 to 0.08; *p* = 0.057), with further attenuation under a 3-mm shift (−2.08 mm; 95% CI, −4.89 to 0.73; *p* = 0.147). In the corresponding covariate-adjusted LVEF model, the overall time effect remained nonsignificant (F(2, 98.35) = 3.06, *p* = 0.051), while the exploratory baseline-to-15-month contrast was +4.41 percentage points (95% CI, 0.86–7.95; nominal *p* = 0.015).

Changes in body weight were not significantly associated with changes in LVEDD from baseline to 3 months (r = 0.007, *p* = 0.959; n = 66) or baseline to 15 months (r = 0.221, *p* = 0.248; n = 29), nor with changes in LVEF from baseline to 3 months (r = 0.030, *p* = 0.834; n = 52) or baseline to 15 months (r = −0.043, *p* = 0.811; n = 33). In a sensitivity analysis among participants with paired baseline and 15-month measurements, LVEDD indexed to body surface area also decreased from baseline to 15 months (mean change, −1.77 mm/m^2^; 95% CI, −2.92 to −0.61; *p* = 0.004; n = 29).

The overall time effect for LVEF was not significant (F(2, 97.22) = 2.67, *p* = 0.074), and neither the dietary group effect (F(1, 101.85) = 2.75, *p* = 0.101) nor the group × time interaction (F(2, 97.22) = 0.36, *p* = 0.698) was significant. The exploratory baseline-to-15-month contrast was +4.11 percentage points (95% CI, 0.55–7.67; nominal *p* = 0.024; Holm-adjusted *p* = 0.072). LVPWT did not change significantly over time (F(2, 115.72) = 1.26, *p* = 0.288), with no significant dietary group effect (F(1, 113.43) = 0.01, *p* = 0.923) or group × time interaction (F(2, 115.72) = 1.90, *p* = 0.155).

**Figure 2:**
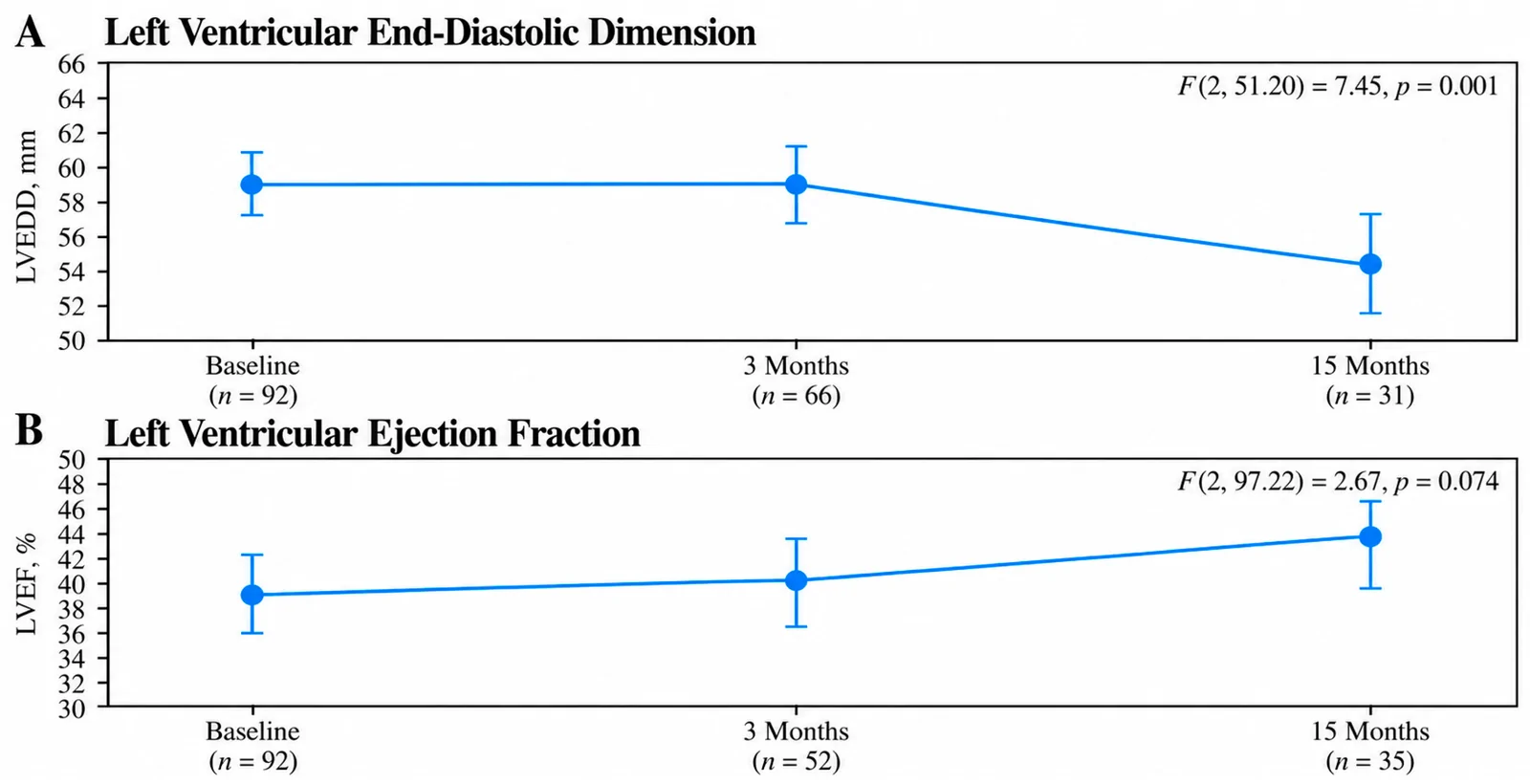
Longitudinal Changes in Left Ventricular Measures Across Study Assessments. Model-estimated marginal means and 95% confidence intervals are shown at baseline, 3 months, and 15 months. Panel (**A**) shows left ventricular end-diastolic dimension (LVEDD), and Panel (**B**) shows left ventricular ejection fraction (LVEF). Participant numbers with available measurements at each assessment are shown beneath the corresponding time points. LVEDD data were available for 92, 66, and 31 participants at baseline, 3 months, and 15 months, respectively; corresponding LVEF data were available for 92, 52, and 35 participants. LVEDD = left ventricular end-diastolic dimension; LVEF = left ventricular ejection fraction.

The longitudinal trajectories for LVEDD and LVEF are illustrated in Figure 2.

The full model-estimated means and phase-specific contrasts are presented in Table 2.

### 3.3. Associations Between Structural and Functional Remodeling

Exploratory correlations between changes in LVEDD and LVEF were not statistically significant. From baseline to 3 months, the association was inverse (r = −0.235, *p* = 0.094; n = 52); from baseline to 15 months, it was small and positive (r = 0.115, *p* = 0.539; n = 31). Spearman analyses produced similar results. Given the limited number of paired observations, particularly at 15 months, these analyses were considered inconclusive. In a covariate-adjusted sensitivity model including sex, race/ethnicity, baseline BMI, and baseline LVPWT, the longitudinal LVEDD finding was essentially unchanged (overall time effect, F(2, 103.20) = 4.76, *p* = 0.011), with an adjusted baseline-to-15-month difference of −3.65 mm (95% CI, −6.10 to −1.21; *p* = 0.004).

## 4. Discussion

In this longitudinal analysis of adults with chronic HF, T2DM, and overweight or obesity, LVEDD decreased over 15 months. LVEDD changed little during the initial 3-month intensive weight-loss phase but was lower at 15 months, with an estimated baseline-to-15-month reduction of 4.44 mm. LVEF did not change significantly over time. Although an exploratory baseline-to-15-month contrast suggested an increase, this contrast did not remain statistically significant after adjustment for multiple comparisons. Remodeling trajectories did not differ between the high-protein and standard-protein groups. Because favorable reverse remodeling has been associated with better outcomes in HF [3], the observed reduction in LV chamber dimension is potentially relevant. The magnitude of the observed baseline-to-15-month LVEDD reduction (4.44 mm) was modest. Because the archived study records did not include intra- or inter-observer reproducibility estimates or sufficient detail on the LV linear measurement method, we cannot determine how much of this change exceeds measurement variability. In the meta-analysis by Kramer et al. [3], a mean reduction of 10 mL in LV end-diastolic volume was associated with a more favorable mortality signal across randomized trials; however, that analysis evaluated volumetric rather than linear measures of LV remodeling. Accordingly, the present change in LVEDD should not be interpreted as quantitatively equivalent to the remodeling effects associated with outcomes in that study.

The temporal pattern of LVEDD change is of particular interest. Most of the estimated reduction occurred after the initial intensive weight-loss phase. Although this may suggest that structural adaptation develops more slowly than the early behavioral and metabolic effects of weight loss, that interpretation remains hypothesis-generating. The completer-only sensitivity analysis produced a similar baseline-to-15-month reduction, supporting the direction and approximate magnitude of the primary finding. The reduction in LVEDD also remained evident after indexing for body surface area, suggesting that the change in chamber dimension was not explained solely by concurrent changes in body size. However, individual changes in body weight were not significantly associated with changes in LVEDD at either follow-up interval, further limiting inference that weight loss itself accounted for the observed remodeling. However, participants with and without 15-month LVEDD measurements differed on several baseline characteristics, and the smaller follow-up sample leaves open the possibility of selective attrition, regression to the mean, or measurement variability. Thus, the findings support a late-emerging reduction in LVEDD but do not establish a delayed biologic remodeling mechanism.

These findings add a dietary perspective to the growing literature on obesity treatment and cardiac remodeling in HF. In STEP-HFpEF, semaglutide improved left atrial and right ventricular remodeling but did not significantly change LV dimensions, mass, or systolic function [5]. In the SUMMIT cardiac magnetic resonance substudy, tirzepatide reduced LV mass and paracardiac adipose tissue in patients with obesity-related HFpEF [14]. Studies on bariatric surgery have also reported favorable changes in LV mass and geometry following substantial weight loss [4]. These studies differ considerably in population, treatment, amount of weight loss, imaging method, and HF phenotype and therefore should not be viewed as directly comparable treatment effects. Together, however, they suggest that favorable cardiac structural changes may accompany obesity treatment, although the specific response varies by intervention and cardiac measure.

Evidence for intentional dietary weight loss in established HF remains much more limited, as shown in the randomized trial by Kitzman et al., in which caloric restriction in older adults with obesity and HFpEF reduced weight and improved peak oxygen consumption but was also associated with loss of lean body mass and thigh muscle area [15]. This highlights the need to consider not only the amount of weight lost but also its composition. Relatively few studies have examined whether changes in cardiac structure accompany structured dietary weight loss [6,7]. Recent guidance continues to emphasize lifestyle and nutritional approaches as foundational components of obesity management in HF, even as anti-obesity pharmacotherapy has expanded treatment options [16]. The present study adds to this evidence by examining serial LV measures during a structured, nonpharmacologic weight-management program.

The absence of a diet group × time interaction for LVEDD, LVEF, and LVPWT indicates that remodeling trajectories did not differ significantly by randomized dietary assignment. Interpretation of these null findings is, however, limited by incomplete dietary data and limited separation in achieved protein intake. Among participants with available 3-day food records, mean protein intake was 80.8 ± 20.6 g/day in the high-protein group and 74.7 ± 24.8 g/day in the standard-protein group; protein accounted for 23.2 ± 8.7% and 23.8 ± 4.0% of energy, respectively. Thus, the achieved protein contrast was substantially smaller than prescribed. The limited separation likely reflects imperfect adherence to the prescribed macronutrient targets, a recognized challenge in behavioral dietary interventions. The parent Pro-HEART trial similarly noted variability in adherence to the assigned diets despite individualized dietitian counseling and repeated adherence support [9]. These findings therefore do not establish that dietary protein content is unimportant for cardiac remodeling; rather, the limited dietary separation reduced the ability of the between-group analyses to detect an independent effect of dietary protein composition.

The relationship between structural and functional remodeling remains uncertain. Although LVEDD decreased over time, the exploratory baseline-to-15-month LVEF contrast did not remain statistically significant after adjustment for multiplicity, and changes in LVEDD were not significantly correlated with changes in LVEF. These exploratory analyses involved relatively small numbers of participants with paired measurements, particularly at 15 months, and had limited ability to detect modest associations. The inverse 3-month correlation was directionally consistent with greater reductions in LVEDD accompanying greater increases in LVEF, but the estimate was imprecise. Larger studies with more complete longitudinal imaging are needed to determine whether changes in LV chamber dimension and systolic function are related during dietary weight management.

Pro-HEART also provides a distinct clinical context from much of the contemporary obesity-treatment literature, which has largely focused on HFpEF. Participants had chronic HF, T2DM, and overweight or obesity, with a mean baseline LVEF of approximately 39%, compared with the preserved ejection fraction populations enrolled in STEP-HFpEF [5] and SUMMIT [14]. Contemporary guidance identifies intentional obesity treatment in patients with reduced systolic function as an important evidence gap [16]. Although this study was not designed to evaluate treatment effects by HF phenotype or to establish efficacy in HFrEF, the cohort’s lower average LVEF supports further study of dietary weight management and cardiac remodeling across a broader HF population.

Several strengths support the present analysis. Pro-HEART was a randomized dietary trial with an intensive weight-loss phase followed by a longer-term maintenance phase, allowing cardiac measures to be examined across clinically meaningful periods of follow-up. Serial echocardiographic assessments provided longitudinal information on LV structure and systolic function, while mixed-effects models incorporated all available repeated measurements. The three assessment points also allowed characterization of the phase-specific pattern of LVEDD change. Attrition and completer sensitivity analyses further assessed the consistency of the primary finding.

The findings should be interpreted in light of several important limitations. Most importantly, the parent trial randomized participants to two dietary compositions rather than to a dietary weight-loss intervention versus a non-weight-loss control condition. Although participants were required to be on stable, optimized HF medical therapy for at least 3 months before enrollment, longitudinal medication data were not available for the present secondary analysis. Changes in HF pharmacotherapy during the 15-month follow-up therefore cannot be excluded as contributors to the observed changes in LVEDD and LVEF. Consequently, the observed longitudinal changes in LVEDD and LVEF cannot be attributed specifically to weight loss or dietary modification. They cannot be distinguished from other potential influences, including regression to the mean, natural disease course, changes in clinical status, or concurrent modification of HF therapy. This is particularly relevant given the reduced mean LVEF at baseline, because changes in evidence-based HF treatment could plausibly contribute to both reductions in LV chamber dimension and improvements in systolic function. The absence of diet group × time interaction further limits inference regarding a specific dietary mechanism.

Second, echocardiographic availability declined substantially over 15 months, particularly for LVEDD and LVPWT. Weight data were also incomplete at 15 months, with measurements available for only 40 participants, limiting interpretation of longer-term weight maintenance and its relationship with cardiac remodeling. Although the completer-only sensitivity analysis produced a baseline-to-15-month LVEDD estimate that was similar in direction and magnitude to the primary model, baseline characteristics differed between participants with and without 15-month LVEDD measurements; therefore, selective attrition and bias due to missing follow-up measurements cannot be excluded. Detailed reasons for the absence of 15-month echocardiographic assessments were not consistently available in the archived analytic dataset, limiting our ability to determine whether loss to follow-up was attributable to clinical deterioration or other causes.

A delta-adjusted sensitivity analysis further showed that the baseline-to-15-month LVEDD finding was no longer statistically significant when unobserved 15-month values were assumed to be approximately 2 mm higher than predicted under the missing-at-random model, indicating that the finding is sensitive to relatively modest departures from that assumption. Although echocardiograms were interpreted by designated cardiologist readers blinded to treatment assignment using standardized study procedures, formal intra- and inter-reader reproducibility data and detailed information regarding sonographer staffing, equipment consistency, and timing relative to fasting or diuretic administration were not available for this secondary analysis.

Third, cardiac assessment was limited to LVEDD, LVEF, and LVPWT. Measures such as LV mass and volumes, global longitudinal strain, diastolic function, left atrial structure, and myocardial tissue characterization would provide a more comprehensive assessment of remodeling. Finally, the exploratory change-score correlations involved relatively small subsets of participants and were insufficient to determine whether structural and functional changes were related.

Overall, the findings demonstrate a longitudinal reduction in LVEDD during the dietary weight-management intervention, with the reduction most apparent at longer-term follow-up. Larger studies with more complete imaging, dietary assessment, body-composition measures, and concurrent HF treatment data are needed to clarify the mechanisms and clinical significance of these changes.

## 5. Conclusions

In adults with chronic HF, T2DM, and overweight or obesity, LVEDD decreased over 15 months, with the reduction most apparent at longer-term follow-up. LVEF did not change significantly over time, and the exploratory baseline-to-15-month contrast did not remain statistically significant after multiplicity adjustment. Remodeling trajectories did not differ by randomized dietary assignment, although limited separation in achieved protein intake constrains interpretation of the between-group findings. Interpretation is further limited by substantial missing 15-month echocardiographic data, with LVEDD available for only 31 of 92 participants, and by the absence of longitudinal HF medication data. Because both groups received hypocaloric diets and there was no non-dietary control group, the observed cardiac changes cannot be attributed specifically to weight loss or the dietary intervention. These findings support further study of longitudinal cardiac remodeling during dietary weight management in HF.

## Figures and Tables

**Table 1:** Baseline Characteristics of the Pro-HEART Cardiac Remodeling Analytic Sample Overall and by 15-Month LVEDD Availability.

Characteristic	Overall (*N* = 92)	15-Month LVEDD Available (*n* = 31)	15-Month LVEDD Unavailable (*n* = 61)	Absolute SMD
Age, years	58.5 ± 9.7	59.6 ± 9.8	57.9 ± 9.7	0.173
Sex, *n* (%)				
Female	28 (30.4)	6 (19.4)	22 (36.1)	0.380
Male	64 (69.6)	25 (80.6)	39 (63.9)	
Race/ethnicity, *n* (%)				
Black	12 (13.0)	6 (19.4)	6 (9.8)	0.272
White	49 (53.3)	13 (41.9)	36 (59.0)	0.347
Asian/Other	17 (18.5)	4 (12.9)	13 (21.3)	0.225
Hispanic	14 (15.2)	8 (25.8)	6 (9.8)	0.427
Randomized dietary group, *n* (%)				
High-protein diet	41 (44.6)	18 (58.1)	23 (37.7)	0.416
Standard-protein diet	51 (55.4)	13 (41.9)	38 (62.3)	
NYHA functional class, *n* (%)				
Class II	69 (75.0)	24 (77.4)	45 (73.8)	0.085
Class III	23 (25.0)	7 (22.6)	16 (26.2)	
Type 2 diabetes mellitus, *n* (%)	92 (100.0)	31 (100.0)	61 (100.0)	—
Body mass index, kg/m^2^	37.2 ± 6.3	35.7 ± 6.5	37.9 ± 6.2	0.343
Baseline LVEDD, mm	58.9 ± 8.0	60.0 ± 9.2	58.4 ± 7.4	0.201
Baseline LVEF, %	38.7 ± 12.7	37.5 ± 13.0	39.3 ± 12.6	0.140
Baseline LVPWT, mm	10.9 ± 1.8	11.2 ± 2.2	10.7 ± 1.6	0.257

Note. Values are mean ± SD or *n* (%), as appropriate. Continuous variables are reported to one decimal place, percentages to one decimal place, and standardized mean differences (SMDs) to three decimals. For binary variables, the SMD is shown on the first category row only. Absolute SMDs compare participants with versus without 15-month LVEDD measurements. NYHA = New York Heart Association; LVEDD = left ventricular end-diastolic dimension; LVEF = left ventricular ejection fraction; LVPWT = left ventricular posterior wall thickness.

**Table 2:** Longitudinal Changes in Body Weight, BMI, and Left Ventricular Measures During the Dietary Weight-Management Trial.

**Panel A.** Model-estimated measures across study assessments.												
**Outcome**	**Baseline,** **EMM (95% CI)**		**3 Months,** **EMM (95% CI)**		**15 Months,** **EMM (95% CI)**		**Time Effect,** **F(df1, df2), *p***		**Group Effect,** **F(df1, df2), *p***		**Group × Time,** **F(df1, df2), *p***	
Body weight, kg	108.90 (104.43–113.37)		105.87 (101.40–110.34)		108.48 (103.92–113.05)		F(2, 128.35) = 20.19 *p* < 0.001		F(1, 90.56) = 1.34 *p* = 0.250		F(2, 128.35) = 2.84 *p* = 0.062	
BMI, kg/m^2^	37.08 (35.73–38.43)		36.08 (34.73–37.43)		36.93 (35.54–38.31)		F(2, 128.40) = 21.22 *p* < 0.001		F(1, 90.64) = 0.85 *p* = 0.360		F(2, 128.40) = 2.89 *p* = 0.059	
LVEDD, mm	59.00 (57.32–60.68)		58.98 (56.78–61.19)		54.56 (51.75–57.37)		F(2, 51.20) = 7.45 *p* = 0.001		F(1, 87.56) = 1.14 *p* = 0.289		F(2, 51.20) = 0.47 *p* = 0.627	
LVEF, %	38.96 (36.21–41.72)		39.78 (36.41–43.15)		43.07 (39.28–46.87)		F(2, 97.22) = 2.67 *p* = 0.074		F(1, 101.85) = 2.75 *p* = 0.101		F(2, 97.22) = 0.36 *p* = 0.698	
LVPWT, mm	10.84 (10.43–11.25)		10.37 (9.82–10.93)		10.59 (9.92–11.26)		F(2, 115.72) = 1.26 *p* = 0.288		F(1, 113.43) = 0.01 *p* = 0.923		F(2, 115.72) = 1.90 *p* = 0.155	
**Panel B.** Phase-specific contrasts.												
**Outcome**	**Baseline → 3 Months**				**3 → 15 Months**				**Baseline → 15 Months**			
	**EMD (95% CI)**	**Nominal *p***		**Holm-Adjusted *p***	**EMD (95% CI)**	**Nominal *p***		**Holm-Adjusted *p***	**EMD (95% CI)**	**Nominal *p***		**Holm-Adjusted *p***
Body weight, kg	−3.03 (−4.01 to −2.05)	<0.001		—	+2.62 (1.25 to 3.99)	<0.001		—	−0.41 (−1.78 to 0.96)	0.551		—
BMI, kg/m^2^	−1.00 (−1.32 to −0.69)	<0.001		—	+0.85 (0.41 to 1.29)	<0.001		—	−0.16 (−0.60 to 0.29)	0.489		—
LVEDD, mm	−0.02 (−1.51 to 1.47)	0.982		0.982	−4.42 (−7.52 to −1.33)	0.006		0.012	−4.44 (−6.86 to −2.02)	<0.001		0.002
LVEF, percentage points	+0.82 (−2.28 to 3.92)	0.601		0.601	+3.29 (−0.51 to 7.09)	0.089		0.178	+4.11 (0.55 to 7.67)	0.024		0.072
LVPWT, mm	−0.47 (−1.06 to 0.12)	0.119		0.357	+0.22 (−0.54 to 0.98)	0.573		0.954	−0.25 (−0.95 to 0.45)	0.477		0.954
**Panel C.** Model-estimated body weight and BMI by randomized dietary group.												
**Measure and Group**		**Baseline,** **EMM (95% CI)**			**3 Months,** **EMM (95% CI)**				**15 Months,** **EMM (95% CI)**			
Body weight, kg												
High-protein		106.13 (99.48–112.79)			102.55 (95.89–109.20)				106.80 (100.02–113.58)			
Standard-protein		111.66 (105.69–117.63)			109.19 (103.22–115.16)				110.17 (104.04–116.30)			
BMI, kg/m^2^												
High-protein		36.42 (34.41–38.43)			35.21 (33.20–37.22)				36.59 (34.54–38.64)			
Standard-protein		37.74 (35.94–39.54)			36.94 (35.14–38.74)				37.26 (35.41–39.12)			

Note. Values are estimated marginal means (EMMs) and 95% confidence intervals from linear mixed-effects models. For LVEDD, an unstructured within-participant covariance structure was retained; for LVEF, LVPWT, body weight, and BMI, random-intercept models were retained. Estimated mean differences (EMDs) are expressed as later minus earlier values. Holm-adjusted *p*-values account for the three phase-specific contrasts within each echocardiographic outcome; body weight and BMI comparisons were contextual analyses and were not included in this multiplicity adjustment. Phase-specific contrasts correspond to the time-point comparisons specified in the parent Pro-HEART trial protocol [8] (baseline to 3 months, 3 to 15 months, and baseline to 15 months); the present echocardiographic secondary analysis was conducted post hoc. Body weight and BMI data were available for 92 participants at baseline, 92 at 3 months, and 40 at 15 months. Echocardiographic measurements were available at baseline, 3 months, and 15 months for LVEDD (n = 92, 66, and 31), LVEF (n = 92, 52, and 35), and LVPWT (n = 92, 50, and 31), respectively. Clinically meaningful weight loss of ≥5% of baseline body weight occurred in 17/92 participants (18.5%) at 3 months and 4/40 participants (10.0%) with available weight data at 15 months. LVEDD = left ventricular end-diastolic dimension; LVEF = left ventricular ejection fraction; LVPWT = left ventricular posterior wall thickness; BMI = body mass index.

## Data Availability

The article includes the original contributions presented in the study; further inquiries may be directed to the corresponding author.

## Abbreviations

The following abbreviations are used in this manuscript:

CI	confidence interval
EMD	estimated mean difference
EMM	estimated marginal mean
HF	heart failure
LV	left ventricular
LVEDD	left ventricular end-diastolic dimension
LVEF	left ventricular ejection fraction
LVPWT	left ventricular posterior wall thickness
T2DM	type 2 diabetes mellitus
